# Lane Following Method Based on Improved DDPG Algorithm

**DOI:** 10.3390/s21144827

**Published:** 2021-07-15

**Authors:** Rui He, Haipeng Lv, Sumin Zhang, Dong Zhang, Hang Zhang

**Affiliations:** State Key Laboratory of Automotive Simulation and Control, Jilin University, Changchun 130025, China; herui@jlu.edu.cn (R.H.); lvhp19@mails.jlu.edu.cn (H.L.); dong_jlu@163.com (D.Z.); hzhang19@mails.jlu.edu.cn (H.Z.)

**Keywords:** deep reinforcement learning, autonomous driving, deep deterministic policy gradient, lane following

## Abstract

In an autonomous vehicle, the lane following algorithm is an important component, which is a basic function of autonomous driving. However, the existing lane following system has a few shortcomings: first, the control method it adopts requires an accurate system model, and different vehicles have different parameters, which needs a lot of parameter calibration work. The second is that it may fail on road sections where the lateral acceleration requirements of vehicles are large, such as large curves. Third, its decision-making system is defined based on rules, which has disadvantages: it is difficult to formulate; human subjective factors cannot guarantee objectivity; coverage is difficult to guarantee. In recent years, the deep deterministic policy gradient (DDPG) algorithm has been widely used in the field of autonomous driving due to its strong nonlinear fitting ability and generalization performance. However, the DDPG algorithm has overestimated state action values and large cumulative errors, low training efficiency and other issues. Therefore, this paper improves the DDPG algorithm based on the double critic networks and priority experience replay mechanism. Then this paper proposes a lane following method based on this algorithm. Experiment shows that the algorithm can achieve excellent following results under various road conditions.

## 1. Introduction

Lane following is one of the most important autonomous driving subsystems. Only after successfully implementing the lane following function can other advanced subsystems of autonomous driving such as obstacle avoidance and car following be further developed [1]. The existing lane following algorithm only considers the lateral motion of the vehicle, and rarely considers the influence of the longitudinal dynamics of the vehicle. However, because the lateral dynamics and longitudinal dynamics of the vehicle are coupled with each other, its applicable occasions are limited to situations with low speed or small steering angles [2]. When the vehicle needs a large steering angle input, its control strategy is difficult to meet the requirements. Such lane following control methods include linear quadratic regulator (LQR), model predictive control (MPC), etc. These methods have higher requirements for system models. Due to different parameters and different models, it is difficult to obtain a universal system model. The decision-making part of the existing lane following system is constructed based on rules. When modeling it, it is necessary to consider a lot of working conditions and adapt suitable solutions for it. This is likely to miss several working conditions that may occur during the vehicle driving process and the manual decision-making methods for various working conditions may not be optimal [3].

In recent years, artificial intelligence technology applied to autonomous driving has developed rapidly, especially reinforcement learning technology [4,5,6,7,8]. The first control example based on reinforcement learning (RL) was inspired by the concept of ALVINN [9]. Like ALVINN, the output of the neural network is a discrete steering angle. The architecture is a simple feedforward network in which raw camera data is used as input. In [10], there is a better concept, which uses the deep Q network algorithm to train a neural network to predict the appropriate yaw acceleration for lane changing operations. The authors of [11] combine reinforcement learning with fuzzy logic to create a hybrid method for vehicle longitudinal control. The RL method is a network of Q estimators while fuzzy inference is Takagi-Sugeno. In [12], an agent based on policy gradient is proposed, which uses a multi-objective reward function to train the agent to complete the control task of cooperative adaptive cruise control. The authors in [13] propose a parameterized batch actor-critic algorithm, which uses policy-based and value-based model-free RL technology to solve the longitudinal control problem. In [14], the author introduced a method based on deep Q learning (DQN), which is specifically used to control the braking system to automatically avoid collisions. In [15], the authors proposed a concept using inverse reinforcement learning (IRL), and they managed to train the agent to learn different driving styles from the demonstration. In addition, a good review of imitation and behavioral cloning methods applied in this field can be found in [16].

Recently, some scholars have tried to use deep reinforcement learning methods to design lane-following system for autonomous vehicles. In [17], a method that combines supervised learning and deep Q learning(DQN) is proposed. The author uses precise ideas to formulate his own algorithm to solve the lateral and longitudinal control tasks at the same time. The control task is formulated as keeping the vehicle on the road. In [18], the author compares DQN and deep deterministic actor-critic (DDAC) algorithms to show the importance of using continuous actions in this control problem. These algorithms have one shortcoming, that is, automatic driving is a continuous control problem, but these algorithms can only be applied to discrete problems, so the control quantity must be discretized, and this process will inevitably lead to the inability to deal with the surrounding environment dynamic factors in the system and imprecise control [19,20,21].

In order to solve this problem, the authors in [22] propose the DDPG algorithm, which is an algorithm based on direct policy search that can directly output continuous action values, which is very suitable for continuous control environments. The author applied it to lane following and achieved good results in the TORCS environment. It combines actor-critic algorithm [23], deterministic policy gradient [24], and DQN algorithm [25]. However, using it directly in autonomous driving scenarios will have the following problems. First, in the DDPG algorithm, each iteration of the actor network will overestimate the Q value. When the number of training increases, the cumulative error will accumulate greatly. At the same time, the inaccurate estimation of the Q value will also lead to the decline of the strategy update effect. The second reason is that each step will produce a small error δ(s, a) during the TD update. When multiple updates are performed, the error will be accumulated in a large amount, resulting in an inaccurate Q value. Since the decision-making and planning layer of autonomous vehicles has higher requirements for the accuracy of the output action values, slight disturbances in the steering or throttle may cause the driving effect to decrease or even accidents [26]. The third is that experience replay is to randomly sample data stored in the experience pool, which will result in low expectations of the sampled samples and slower training [27]. For autonomous driving, a project that requires a lot of manpower and material resources will cause much waste of resources.

For dealing with this problem, this paper proposes double critic networks and priority experience replay deep deterministic policy gradient (DCPER-DDPG) algorithm. First, this paper proposes a lane following algorithm architecture based on deep reinforcement learning; secondly, designs the reward function, exploration strategy, and improved DDPG algorithm; finally, the algorithm proposed in this paper is tested and verified on the TORCS simulation platform.

## 2. Algorithm Architecture

### 2.1. Lane Following Algorithm Framework

**Figure 1:**
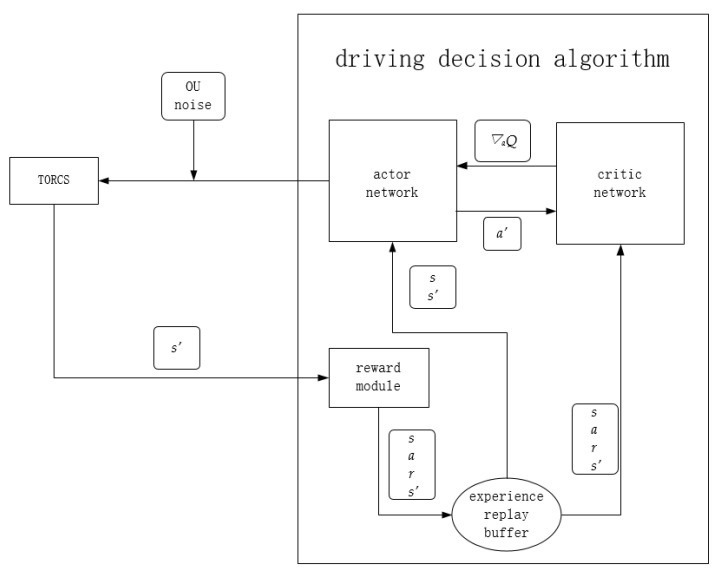
Lane following algorithm framework.

The general framework of the lane following algorithm adopted in this paper is shown in Figure 1. The execution process of the algorithm is as follow:

- A set of action values is generated by the actor network, namely steering, throttle, and braking values, and random noise is added and input into the TORCS simulation software;
- TORCS input the next state *s*’ into the reward function module according to the action, and store it into the experience pool together with the current state *s*, reward value *r*, and action value *a*;
- Sample a certain number of samples from the experience replay buffer, each sample contains *s*, *a*, *r*, *s*’. Then respectively pass *s*, *s*’ to the actor network, and *s*, *a*, *r*, *s*’ to the critic network for an iterative update;
- For the actor network, it accepts *s* and *s*’, then outputs *a* to TORCS together with random noise. Meanwhile, it outputs the next action *a*’ to the critic network. After that, accepts the gradient of the state action value *Q*(*s*, *a*) to *a*’ to update the network.
- For the critic network, it accepts *s*’, *a*’ to calculate *Q*(*s*’, *a*’). Next, it combines *Q*(*s*’, *a*’) with *r* to calculate a label for the iterative update of the network. At the same time, *s* and *a* are input into the critic network, and the mean square error between the output and the label is used as the loss function to update the network iteratively. Finally, calculate the gradient of the *Q* value to *a*’ under *s* and return that to the actor network for an iterative update.

### 2.2. Input Data Selection

The input of the algorithm is composed of environmental information obtained by a variety of sensors. The environmental information includes the longitudinal speed of the vehicle, the lateral speed of the vehicle, the angle between the vehicle heading and the lane, the distance between the vehicle and the centerline of the road, and the distance between the vehicle and the edge of the road. The specific definitions are shown in Table 1.

### 2.3. Network Structure

The overall framework design of the actor network and critic network refers to [28].

#### 2.3.1. Actor Network Structure

**Figure 2:**
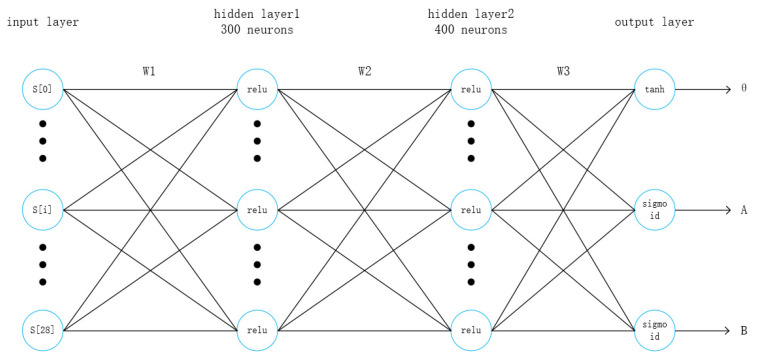
Actor network structure.

The sensor input data type in this article only has low-dimensional data and no picture input, so the actor network does not use a convolutional layer, and its architecture is shown in Figure 2.

The network includes 4 layers, which are 1 input layer, 2 hidden layers, and 1 output layer. The input layer contains 29 neurons, which respectively represent the 29 sensor state values given by the TORCS environment. In [28] the two hidden layers include 300 and 600 neurons respectively, and each neuron uses relu as an activation function. This paper changes the number of neurons in the second hidden layer to 400 in order to reduce overfitting. The output layer contains 3 neurons, which output the three values of steering, throttle, and braking respectively.

Among them, the neuron that outputs the steering value uses tanh as the activation function, and the value range is [−1, +1]. When the output value is +1, it means that the vehicle’s left-turning motion is the largest, and when it is −1, it means the right-turning motion is the largest. The neurons that output throttle and brake both use sigmoid as the activation function, and the value range is [0, 1]. When the output throttle action value is 1, it means that the maximum acceleration is output, and when it is 0, it means that the vehicle is not accelerating at this time. When the output brake value is 1, it means that the output is the maximum deceleration action, and when it is 0, it means that no deceleration action is performed.

#### 2.3.2. Critic Network Structure

**Figure 3:**
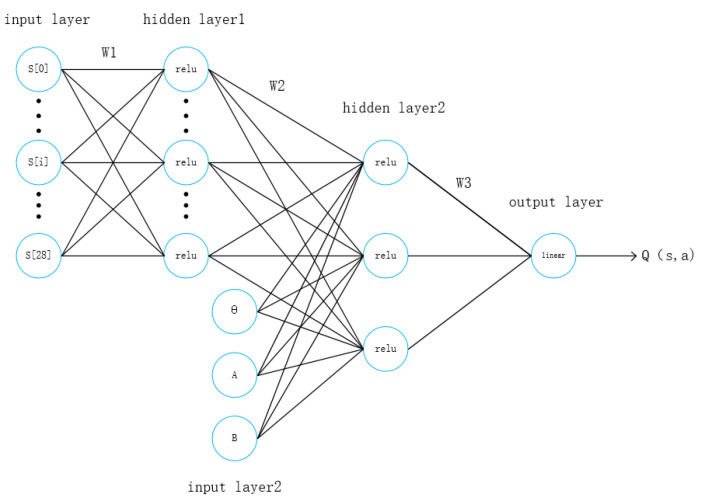
Critic network structure.

The structure of the critic network is shown in Figure 3. The input layer is divided into two parts, one is the environment state information given by the TORCS software, and the other is the action value output by the actor network. The environmental state information is processed by hidden layer 1 and combined with the action value to be input into hidden layer 2, the two layers also include 300 and 400 neurons respectively. Then state information is processed by output neurons to obtain the state action value.

## 3. Lane Following Strategy

### 3.1. Reward Function

In the reinforcement learning algorithm, since the initialization of the network is random, the agent can only interact with the environment under the guidance of the reward function. The reward function is generally a scalar, a positive value represents a reward, and a negative value represents a penalty. The reinforcement learning algorithm is to optimize in the direction that can maximize the reward value. The selected working condition in this paper is lane following so the vehicle is required to stay in the center of the lane as much as possible and the vehicle’s forward direction is as consistent as possible with the road axis, and at the same time, increase the speed as much as possible on this basis. The reward function used in this article is as follows:

(1) $$r=\{\begin{array}{l} -200,\;\;\mathrm{the}\;\mathrm{vehicle}\;\mathrm{drives}\;\mathrm{off}\;\mathrm{the}\;\mathrm{road}\\ \;\;\;v_{x}cos\omega -v_{x}sin\omega -v_{x}\left|trackpos\right|,\;\mathrm{the}\;\mathrm{vehicle}\;\mathrm{drives}\;\mathrm{normally}\;\mathrm{on}\;\mathrm{the}\;\mathrm{road} \end{array}$$

where $v_{x}$ is the speed of the vehicle along the longitudinal axis of the vehicle, ω is the angle between the direction of the vehicle and the axis of the road, and trackpos is the normalized distance between the vehicle and the lane center. The reward function is expected to maximize the axial speed of the vehicle and let the vehicle drive along the road axis.

### 3.2. Exploration Strategy

In deep reinforcement learning algorithms, appropriate exploration strategies must be set to prevent the algorithm from falling into a local optimum. Common exploration strategies such as ε-greedy are not suitable for autonomous driving scenarios. As we have three combined actions (steering, accelerator, braking), if we just randomly select actions from a uniform distribution, some useless combinations may be generated. For example, the value of braking is greater than the value of acceleration, so that the vehicle cannot drive. Therefore, here we use the Ornstein–Uhlenbeck process to add noise:

(2) $$dx_{t}=\zeta \left(\mu -x_{t}\right)dt+\sigma dW_{t}$$

In the formula: ζ represents the speed at which the response variable returns to the mean, μ represents the balance or average, σ represents the degree of fluctuation of the process, and $W_{t}$ is the Wiener process. The values of ζ,  μ, and σ in this process are shown in Table 2.

### 3.3. Double Critic Networks and Priority Experience Replay of DDPG Algorithm

In order to solve the problems of over-estimation of the *Q* value, large accumulated error, and slow training speed of the DDPG algorithm, this paper adopts the following steps to design DCPER-DDPG algorithm:

(1) *Q* value is overestimated

The target value used by the two critic networks for updating can be written as:

(3) $$y_{1}=r+\gamma Q_{\theta_{2}^{\prime }}\left(s^{\prime },\;\pi_{\varphi_{1}}\left(s^{\prime }\right)\right)$$

(4) $$y_{2}=r+\gamma Q_{\theta_{1}^{\prime }}\left(s^{\prime },\;\pi_{\varphi_{2}}\left(s^{\prime }\right)\right)$$

where r is the current reward value, γ is the discount factor, $Q_{\theta_{1}^{\prime }}$ and $Q_{\theta_{2}^{\prime }}$ are the *Q* values calculated by the two critic target networks respectively, $\pi_{\varphi_{1}}$ and $\pi_{\varphi_{2}}$ are the action values calculated by two actor networks, respectively.

The two *Q* values will always appear one large and one small, and the high value will be overestimated. Therefore, choose the minimum value as the final *Q* value.

(5) $$y=r+\gamma \underset{i=1,\;2}{\min}Q_{\theta_{i}^{\prime }}\left(s^{\prime },\;\pi_{\varphi_{i}}\left(s^{\prime }\right)\right)$$

(2) Accumulative error processing

First, the actor network and the target network are updated with a delay. That is, the critic network is updated a certain number of times and then the actor network and the target network are updated.

(3) Speed up training

This paper measures the priority of the sample according to the TD error of the sample. The sample priority expression is as follows:

(6) $$P\left(i\right)=\frac{p_{i}^{\alpha }}{\sum_{k}p_{k}^{\alpha }}$$

This paper adopts the proportional prioritization method to calculate$\;p_{i}$, that is, $p_{i}=\left|\delta_{i}\right|+\epsilon$, where ϵ is a small number to prevent the unsampled state from being given priority to 0, δ is TD error, α determines how much priority is used, when α is 0, it is uniform sampling.

This sampling method introduces bias because it changes the data distribution, which in turn changes the expected value. Thus this paper uses importance sampling to modify this weight:

(7) $$w_{i}={\left(\frac{1}{N}\cdot \frac{1}{P\left(i\right)}\right)}^{\beta }.\;$$

where *N* is the size of batch, and β is a parameter that is between 0 and 1.

When calculating the critic network later, it is multiplied by the importance sampling parameter to adjust the weight.

The improved algorithm is shown in Algorithm 1.
**Algorithm 1: DCPER-DDPG**Randomly initialize the actor network $\pi_{\varphi }$ and critic networks $Q_{\theta_{1}}$ and $Q_{\theta_{2}}$
Initialize the weights of the target networks $\theta_{1}^{\prime }\leftarrow \theta_{1}$,$\;\theta_{2}^{\prime }\leftarrow \theta_{2}$,$\;\varphi^{\prime }\leftarrow \varphi$ Initialize the replay buffer R, Set the maximum storage capacity of the buffer S Initialize state S_0_, batch_size = K Initialize maximum priority D, priority parameters α, β for episode = 1,…, M do: Initialize random noise ε_t_ for t = 0,…, T do: Select actions based on the actor network and add random noise $a_{t}=\pi_{\varphi }\left(s_{t}\right)+\varepsilon_{t}$ and get the next state given by the environment *s_t_*_+1_, reward *r_t_* Store (s_t_, a_t_, r_t_, s_t+1_) into replay buffer R, and set the maximum priority $D_{t}=max_{i<t}D_{i}$  if t > S:   for j = 1, K do:    Sample transition according to priority:(*s_j_*, *a_j_*, *r_j_*, *s_j_*_+1_)    Calculate the optimization goal of the critic network:     $y=r+\gamma \underset{i=1,\;2}{\min}Q_{\theta_{I}^{\prime }}\left(s^{\prime },\;\pi_{\varphi_{i}}\left(s^{\prime }\right)\right)$    Calculate the corresponding importance sampling weight *W_j_* and TD error *δ_j_*    According to the absolute value of TD error |*δ_j_*| update the priority of transition  end for  Update the two critic networks separately by minimizing the loss function:         $L=\frac{1}{K}\underset{i}{\sum }w_{i}\delta_{i}^{2}$  if t mod policy_update_frequency == 0:   Use policy gradient to update the actor network:             $\nabla_{v}\pi {|}_{s_{i}}\approx \frac{1}{K}\underset{i}{\sum }\nabla_{a}Q\left(s_{t},a_{t}\right){|}_{s=s_{i},a=\pi_{\varphi }\left(s_{i}\right)}\nabla_{\varphi }\pi \left(s_{t}\right)|s_{i}$   Use update rate γ to update the weight of the target network $Q_{\theta_{1}^{\prime }}$, $\;Q_{\theta_{2}^{\prime }}$ and $\;\pi_{\varphi^{\prime }}$  end if  end if end for end for

## 4. Experiment

### 4.1. Simulation Environment

The simulation experiment in this paper is carried out on the TORCS platform. TORCS is a highly portable and open-source game platform, which is popular in fields such as intelligent control. The platform provides accurate vehicle dynamics models and different maps, which can better simulate real scenes. This paper uses Python-based Gym-TORCS as the platform and interface with TORCS to control vehicle movement in real time [29].

### 4.2. Termination Condition Setting

In the process of smart car training, there may be scenes that are not conducive to the convergence of the algorithm, such as the vehicle not moving or driving in the opposite direction. Intervention can be made to improve the convergence speed during simulation.

(1) The vehicle stalls. If the longitudinal speed of the target vehicle is always less than 5 km/h in 100 time steps, it will end the current episode and start a new episode.
(2) The vehicle drives off the track. If the vehicle runs off the track, it will automatically end the current episode and restart a new episode.
(3) The vehicle travels in reverse. If the vehicle’s forward direction reverses, a new episode is started.

### 4.3. Scene Selection

**Figure 4:**
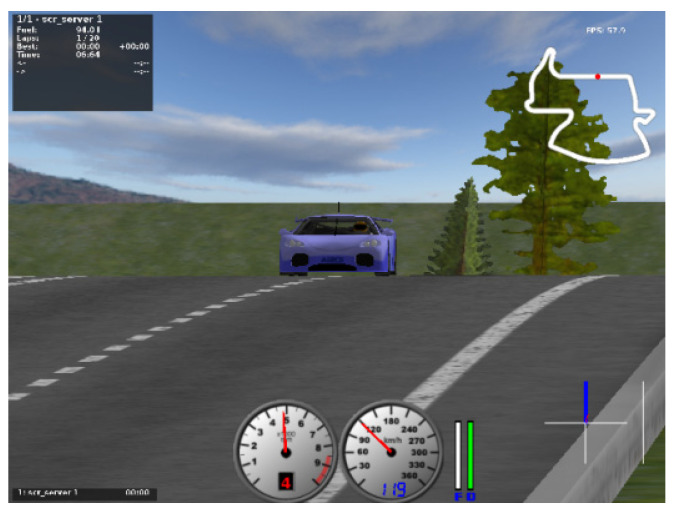
Aalborg map.

**Figure 5:**
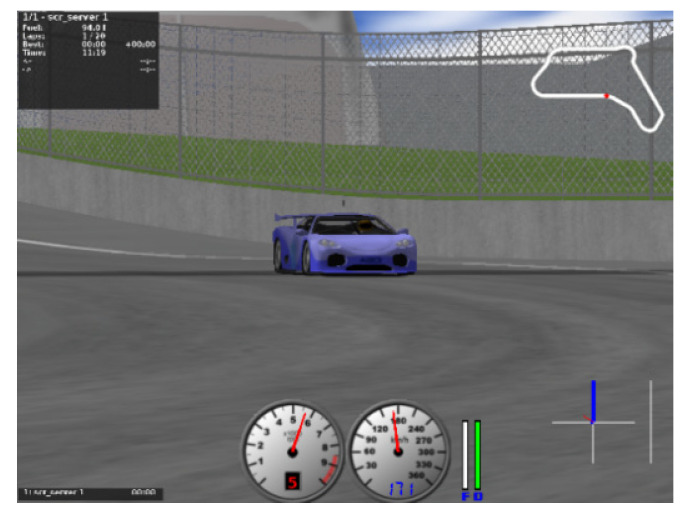
CG track 2 map.

This paper chooses Aalborg as the training track, as shown in Figure 4. The map has a total length of 2587.54 m and a width of 10 m. The typical features of the track are obvious, such as lane lines and static obstacles. The length of the straight line of the Aalborg track is appropriate, and the curvature of the curve is large, which is suitable for verifying the deep reinforcement learning algorithm;

Since the road features of CG track 2 are similar to that of Aalborg, which is shown in Figure 5, this paper chooses it as the test track.

### 4.4. Training Parameter Settings

The network training parameter settings of this paper are shown in Table 3.

## 5. Result Analysis

### 5.1. Training Average Reward

**Figure 6:**
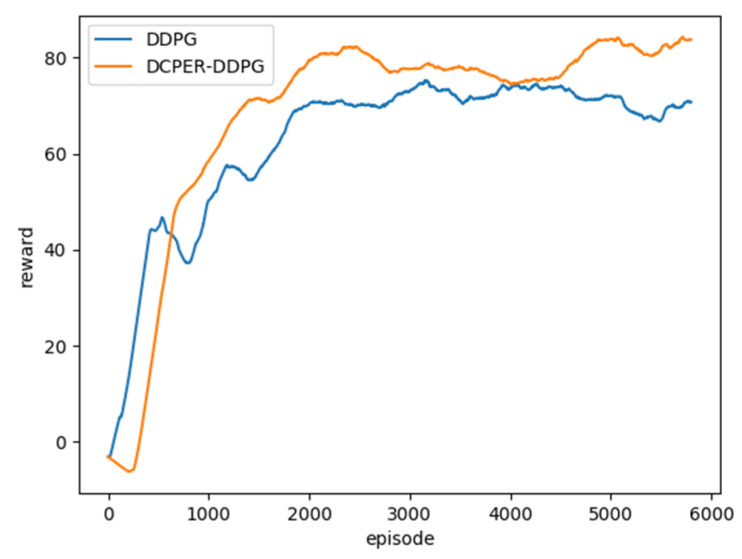
Comparison of single-step average reward value between DDPG and DCPER-DDPG algorithm.

In deep reinforcement learning, the single-step average reward value of each episode is an important indicator to measure the training effect [30,31,32,33]. This paper counts the average single-step rewards of [22] and DCPER-DDPG algorithm in 6000 episodes. Figure 6 is a comparison of the two after the data is Gaussian smoothed.

It can be seen from Figure 6 that due to the delayed update of the actor network in the DCPER-DDPG algorithm, the reward value of the previous training period is lower than that of the original algorithm. However, since about the 800th episode, the reward value of DCPER-DDPG algorithm has exceeded the original algorithm, indicating that the algorithm in this paper has a stronger learning ability and a more stable training process.

### 5.2. Number of Steps Completed in One Episode

In the deep reinforcement learning training process, the vehicle continuously explores the environment, and each exploration is called a “step”. When a vehicle commits an illegal behavior, the current episode of training is terminated. The more steps in each episode, the further the vehicle runs. Therefore, the number of steps completed in each round is also an important indicator for judging the speed and effectiveness of driverless vehicle learning.

**Figure 7:**
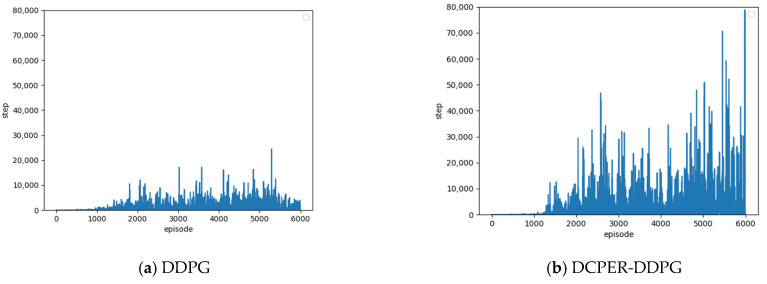
Comparison of Steps between DDPG and DCPER-DDPG.

Figure 7 is the number of steps completed in the episode of DDPG algorithm in [22] and DCPER-DDPG algorithm. By comparing the two figures, it can be seen that the number of steps completed in each episode of the improved algorithm is significantly greater than that of the original algorithm, indicating that the improved algorithm runs farther in each round and has a lower probability of violations.

### 5.3. Analysis of Comparative Results

Put the trained model into the original track to collect the reward value, longitudinal speed, the angle between the vehicle heading, and the lane and the distance between the vehicle and the centerline of the road within 1000 time steps and then calculate their average. The results of comparing the two algorithms are shown in Table 4.

Due to the many corners of the Aalborg map, the vehicle must decelerate when passing the curve, so the improved algorithm speed is only slightly higher than the DDPG algorithm. Since the value of the reward in the setting of our reward function is largely dependent on the vertical speed, the increase is relatively small, about 13%. However, the most important driving performance along the road in the lane following task has been greatly improved. For example, the angle with the lane axis is reduced by 40%, and the distance from the road centerline is reduced by 49%, indicating that the algorithm in this paper is processing the lane following task is significantly better than the DDPG algorithm.

In order to avoid the original model only remembering the features of the original map and failing on another map, we put the model into the CG track 2 track for testing. The test results are shown in Table 5.

CG track 2 has more straight roads, so the speed and reward function of the improved algorithm have been greatly improved compared with [22]. In the comparison of the angle with the lane, the improved algorithm reduced by 81%, the effect was greatly improved, and the distance from the center of the road was also greatly reduced. It can be seen that the DCPER-DDPG algorithm has a stronger generalization ability than the original DDPG algorithm in lane following tasks.

### 5.4. Vehicle Characteristics under the Control of Deep Reinforcement Learning Model

**Figure 8:**
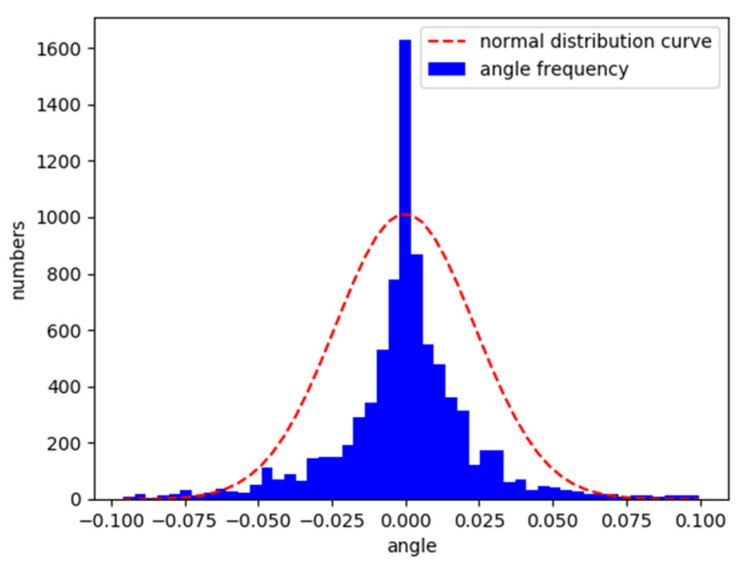
Normalized vehicle and lane centerline angle distribution in Aalborg.

**Figure 9:**
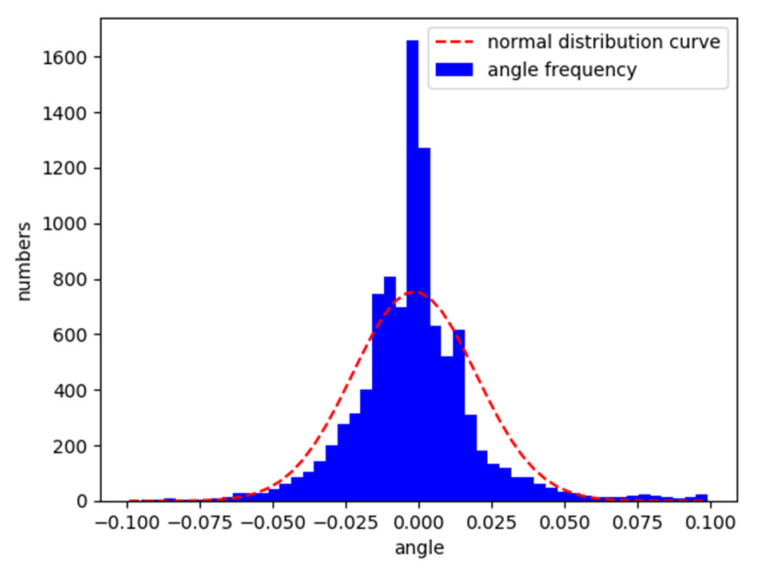
Normalized vehicle and lane centerline angle distribution in CG track 2.

After the training on the Aalborg map, the vehicle was placed on the original map and the CG track 2 map to test its lane following ability. Figure 8 and Figure 9 are the characteristic distributions of the angle between the vehicle’s driving direction and the lane centerline in Aalborg and CG track 2, respectively.

According to the analysis in Figure 8 and Figure 9, the trained model can follow the lane well. The angle between the driving direction of the vehicle and the centerline of the lane is mostly within the range of −0.05 to 0.05. The angle is approximately normally distributed as a whole.

**Figure 10:**
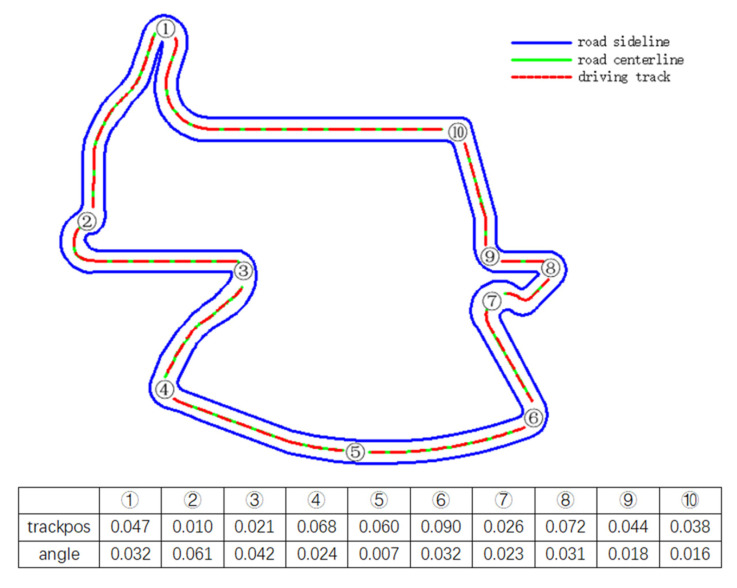
Schematic diagram of lane following of the trained DRL controller vehicle in Aalborg.

**Figure 11:**
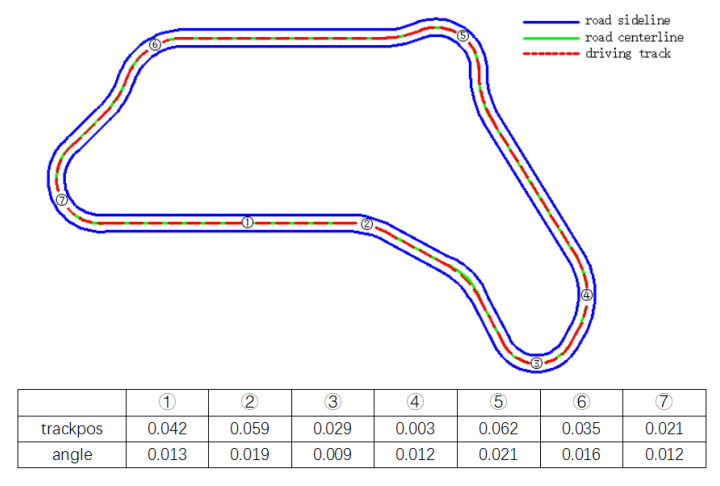
Schematic diagram of lane following of the trained DRL controller vehicle in CG track 2.

Figure 10 and Figure 11 show the lane keeping of the vehicle in CG track 2 more intuitively.

It can be seen from Figure 10 and Figure 11 that whether it is on a straight road or a big curve like ⑧ in Figure 10, the vehicle can maintain a small lateral offset, that is, stay in the center of the lane better. Moreover, the driving direction of the vehicle basically coincides with the axis of the road, showing good lane following performance. Especially in big curves, its lane following performance is basically similar to that of straight lines, which overcomes the shortcoming of the existing lane following system that failure in big curves.

## 6. Conclusions

This paper proposes a lane following method based on the DCPER-DDPG algorithm, designs the input and output, reward function, and exploration strategy of the lane following method, and improves the DDPG algorithm which is widely used in the field of autonomous driving to meet the requirement of lane following situation. Subsequently, the method was experimentally verified under the TORCS platform. The experimental result shows that the reward value and the number of steps in each episode of the DCPER-DDPG algorithm during the training process are higher than those of DDPG, and the trained model also shows that whether lateral deviation or the angle between the vehicle heading and the lane is better than those of DDPG. In terms of lane keeping performance, this method has the characteristics of fewer parameters and strong generalization ability, and its performance is also excellent in large curves, which overcomes the defects of the existing lane keeping systems.

## Figures and Tables

**Table 1:** Definition of selected sensing signals.

Name	Range (Unit)	Description
speedX	(−∞, +∞) (km/h)	Vehicle speed along the longitudinal axis of the vehicle (the direction of driving)
speedY	(−∞, +∞) (km/h)	Vehicle speed along the transverse axis of the vehicle
angle	[−π, +π] (rad)	The angle between the direction of the vehicle and the direction of the road axis
trackpos	(−∞, +∞)	The distance between the car and the road axis, this value is normalized by the width of the road, 0 means the car is on the central axis, and greater than 1 or less than −1 means the car runs off the road
track	(0, 200) (m)	A vector of 19 rangefinder sensors, each sensor returns the distance between the vehicle and the edge of the road within 200 m

**Table 2:** OU process parameter values.

	*ζ*	*μ*	*σ*
Steer	0.60	0.00	0.30
Throttle	1.00	0.50	0.10
Brake	1.00	−0.10	0.05

**Table 3:** Training parameter settings.

Parameter	Value
Buffer size	100,000
Batch size	32
Discount factor	0.99
Soft update factor	0.001
Actor network learning rate	0.0001
Critic network learning rate	0.001
Max steps	100,000
Delayed policy update frequency	2

**Table 4:** Test results of Aalborg map.

	DDPG	DCPER-DDPG
Reward	75.38	85.82
Speed (km/h)	97.15	100.86
Angle (rad)	0.00042	0.00030
Trackpos	0.037	0.019

**Table 5:** Test results of CG track 2 map.

	DDPG	DCPER-DDPG
Reward	87.69	101.98
Speed (km/h)	127.62	140.58
Angle (rad)	0.0029	0.00054
Trackpos	0.13	0.09
